# Amlodipine Overdose in a Transgender Woman: A Case Study

**DOI:** 10.7759/cureus.42511

**Published:** 2023-07-26

**Authors:** Srikaran Bojja, Nismat Javed, Shreya Bojja, Vikram Itare, Rabih Nasr

**Affiliations:** 1 Internal Medicine, BronxCare Health System/Icahn School of Medicine at Mount Sinai, New York, USA; 2 Medicine, Malla Reddy Institute of Medical Sciences, Hyderabad, IND; 3 Nephrology, BronxCare Health System/Icahn School of Medicine at Mount Sinai, New York, USA

**Keywords:** transgender female, tachycardia, hypotension, amlodipine, drug overdose, cardiovascular toxicity, calcium channel blockers

## Abstract

Calcium channel blockers are a major cause of cardiovascular toxicity. The clinical spectrum of these patients is very variable and there is no consensus on the dose required for toxicity. We present a case of a 43-year-old transgender woman who presented with hypotension and tachycardia owing to drug overdose that was later confirmed to be amlodipine. Given the catastrophic cascade of events involved with such toxicities, it is important to highlight amlodipine as one of the causes of drug overdose that can be overlooked.

## Introduction

Calcium channel blockers (CCBs) are one of the common causes of cardiovascular drug toxicity with annual rates as high as 18% [1]. According to the literature, about 8,396 deaths have been reported owing to calcium channel blocker overdose [2]. The overdose can result in detrimental effects including refractory hypotension and shock [3]. Therefore, management of these cases of CCB overdose is particularly challenging [4]. We report a case of a 43-year-old transgender woman who presented with amlodipine toxicity and unfortunately, passed away despite resuscitative efforts. 

## Case presentation

A 43-year-old transgender woman with a past medical history of HIV infection on therapy and hypertension was brought into the emergency room after she took a handful of her anti-hypertensive medication which was later revealed as amlodipine 10 mg tablets. She had taken about 20 to 30 tablets. She had taken these tablets about two hours prior to symptom onset. She did not have a history of surgical procedures. She was not on any hormone replacement therapy as per prior records. Family history was not significant for any diseases. En route to the hospital, the patient was noted to be hypotensive with a blood pressure of 63/49mmHg measured via a digital sphygmomanometer, radial pulse rate of 90/min, and respiratory rate of 16/min. She responded well to 2 liters of 0.9% normal saline bolus initially with mean arterial pressure improving to 65 mmHg. 

In the emergency room, her vitals revealed a temperature of 97.2 degrees F, a pulse rate of 98 beats per minute, a respiratory rate of 22/minute, a blood pressure of 81/51 mmHg, and an oxygen saturation of 99% on room air. Her physical examination revealed altered mentation and low-volume pulse. However, the patient was able to swallow, speak and follow commands. Additionally, the examination was significant for male body habitus. Initial lab investigations are demonstrated in Table 1. Lab investigations were significant for anemia, elevated anion gap metabolic acidosis with lactic acidosis, and increased serum creatinine. These changes were suggestive of a low volume and hypoperfusion state. Urine toxicology did not reveal any source of overdose.

**Table 1:** Lab investigations pCO2: partial pressure of carbon dioxide; pO2: partial pressure of oxygen; NT ProBNP: N-terminal pro–B-type natriuretic peptide

Variable	Value	Reference Value
pH	7.209	7.350-7.450
pCO2 (venous blood gas)	42.3 mmHg	35.0-45.0 mmHg
pO2 (venous blood gas)	41.7 mmHg	83.0-108.0 mmHg
Bicarbonate	16.2 mmol/L	22.0-28.0 mmol/L
Lactic Acid	8.9 mmol/L	0.5-1.6 mmol/L
Serum Sodium	136 mEq/L	135-145 mEq/L
Serum Potassium	4.3 mEq/L	3.5-5.0 mEq/L
Serum Chloride	100 mEq/L	98-108 mEq/L
Blood Urea Nitrogen	19 mg/dL	8.0-26.0 mg/dL
Serum Creatinine	2.1 mg/dL	0.5-1.5 mg/dL
Hemoglobin	10.7 g/dL	12.0-16.0 g/dL
WBC	6.8 K/uL	4.8-10.8 K/uL
Platelets	141 K/uL	150-400 K/uL
NT ProBNP	996 pg/mL	0-125 pg/mL

**Figure 1:**
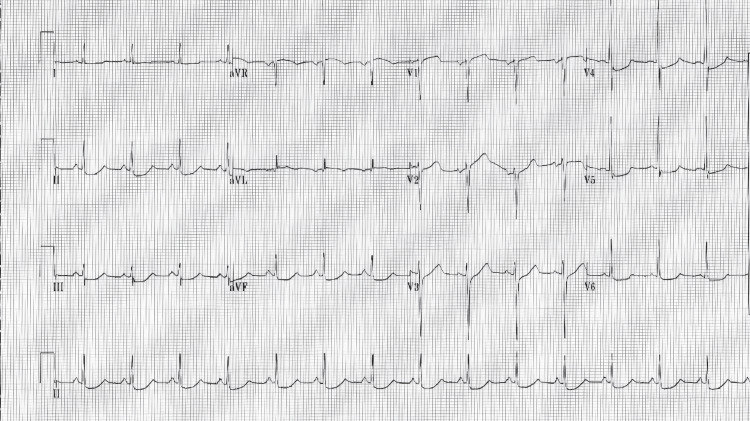
Electrocardiogram at presentation

**Figure 2:**
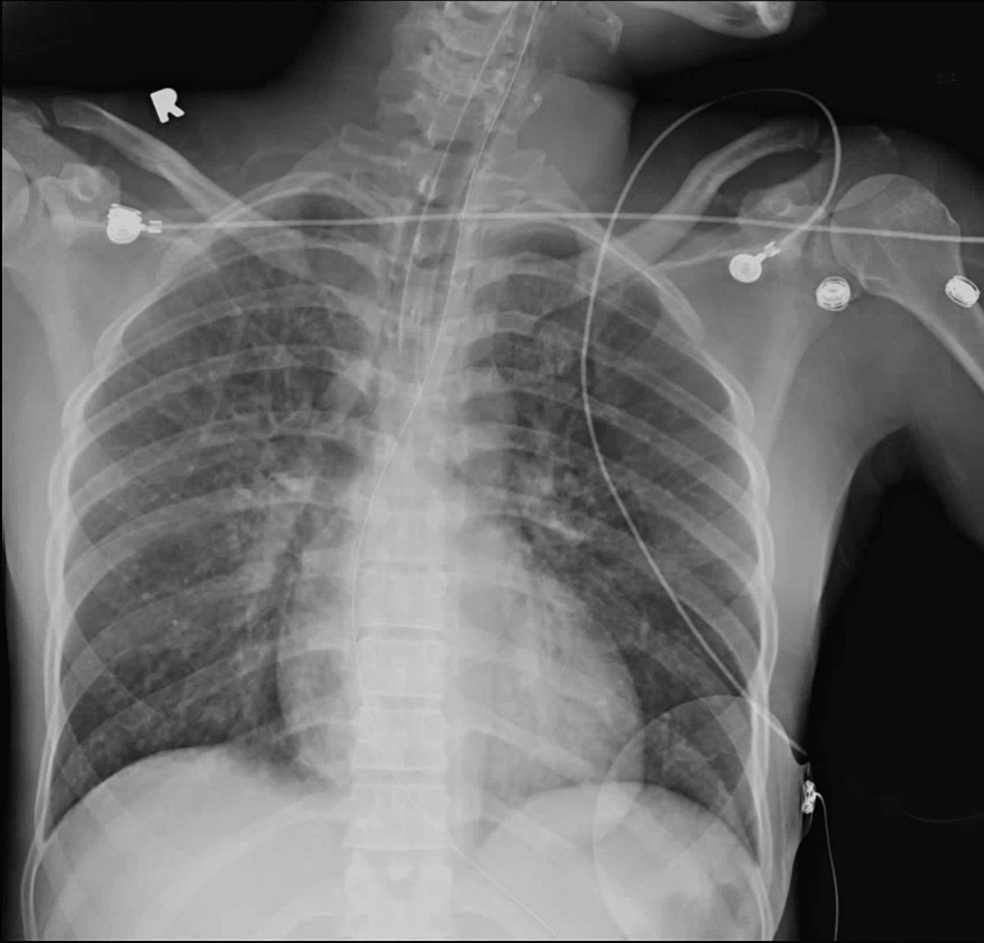
Chest X-ray after intubation (posteroanterior view)

ECG was significant for prolonged QT interval of 486 ms suggestive of negative inotropic impact on the heart (Figure 1). Activated charcoal and sorbitol were administered orally. She received two more liters of 0.9% normal saline fluid bolus with no lasting response. She was also given 2 g of intravenous magnesium sulfate and 1 g of calcium gluconate over one hour. She became more lethargic and therefore was intubated via endotracheal route and transferred to intensive care for further management. Chest X-ray revealed pulmonary congestion (Figure 2). 

The patient was started on hyperinsulinemic-euglycemic protocol [4]. She was started on epinephrine via central line. The patient’s condition did not improve, and she became progressively bradycardic (pulse rate was about 35 beats per minute) and hypotensive (mean arterial pressure ranged from 35 to 40 mmHg). Her condition progressively deteriorated and she became pulseless. After the Advanced Cardiovascular Life Support (ACLS) protocol, the return of spontaneous circulation (ROSC) was achieved after 20 minutes. She became pulseless again after two minutes and died of cardiac arrest within three hours of presentation. 

## Discussion

**Figure 3:**
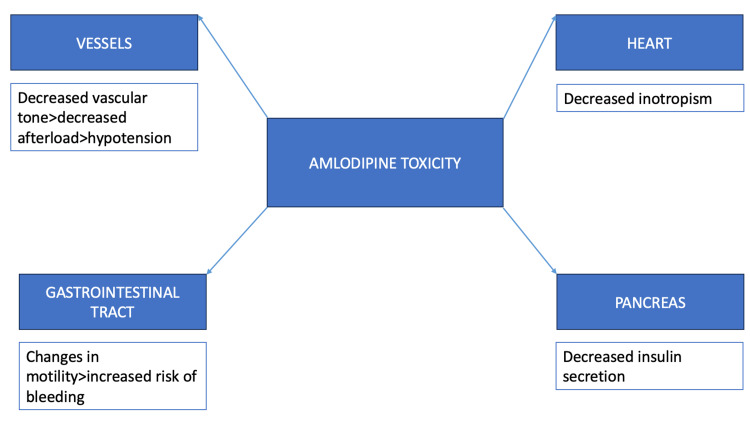
Mechanisms of amlodipine toxicity Image credit: Nismat Javed, Srikaran Bojja

The pharmacokinetics of amlodipine pose a high risk of toxicity. The drug has a relatively longer half-life of about 30-58 hours [5]. Usually, amlodipine is preferred in therapeutic doses due to its greater affinity for smooth muscle as compared to myocardium [6]. However, selective action is lost in cases of toxicity [6,7]. The mechanisms are illustrated in Figure 3. Few cases of amlodipine toxicity have been reported previously [8-10]. 

The age of such patients is quite variable, ranging from as young as 15 years of age to 72 years of age [8,9]. Both male and female gender are at risk with cases being reported in both genders [4,10]. Risk factors for overdose include mood disorders, anxiety, and depression disorder [10]. No cases have been reported of amlodipine toxicity in transgender patients. 

The spectrum of presentation varies from refractory hypotension to atypical presentations including pulmonary edema [10,11]. Hemodynamic instability is a relatively common feature observed in these cases [4,10,11]. Lab investigations are relatively non-specific in this regard. Lactic acidemia might be present but overall labs might be unrevealing [4] as in our case. ECG can be significant for bradycardia, junctional rhythm, third-degree heart block, or even tachycardia in a few cases [12]. 

The exact dose manifesting as an overdose differs according to the patient's age and gender. A few cases have documented doses of about 80 mg to manifest as long-term anoxic brain damage [13]. Interestingly, doses as high as 350 mg have also been reported with patients having a favorable outcome after treatment [12]. 

The management of amlodipine overdose poses a challenge. Treatment primarily focuses on supportive measures to counteract the effects of CCB and enhance intracellular uptake of potassium. In this regard, a hyperinsulinemic-euglycemic protocol has been of immense importance [14,15]. However, this protocol was not effective in our case. Another therapeutic option is intravenous calcium gluconate. However, both positive and negative impacts have been observed. An excessively high calcium infusion might result in pulmonary edema requiring hemodialysis whereas inadequate infusion might fail to reverse the overdose [10,16]. Hemodialysis might not be a suitable option given the avidity of amlodipine to bind to proteins [16]. 

While vasopressors form the mainstay of management in cases of cardiogenic shock, a cohort of patients presenting with refractory shock can be candidates for extracorporeal membrane oxygenation (ECMO). However, it is important that multiorgan damage be recognized early on to allow a larger window for ECMO therapy [13,17]. 

Mortality rates with amlodipine have been significantly high with long-lasting consequences including anoxic brain injury [12,13]. However, no clear relationship between the plasma concentration of amlodipine and mortality has been noted [13]. 

## Conclusions

Amlodipine toxicity can be a life-threatening condition with a high mortality rate that requires prompt recognition and management. As amlodipine cannot be tested for in blood readily, identification of the offending agent should be elicited as soon as possible to ensure directed therapy. Physicians should have a very high suspicion for amlodipine toxicity in cases of refractory hypotension. Healthcare providers need to be vigilant about the groups at high risk of amlodipine toxicity for timely management. 
